# Identification of Odorant-Binding Proteins (OBPs) and Functional Analysis of Phase-Related OBPs in the Migratory Locust

**DOI:** 10.3389/fphys.2018.00984

**Published:** 2018-07-20

**Authors:** Wei Guo, Dani Ren, Lianfeng Zhao, Feng Jiang, Juan Song, Xianhui Wang, Le Kang

**Affiliations:** ^1^State Key Laboratory of Integrated Management of Pest Insects and Rodents, Institute of Zoology, Chinese Academy of Sciences, Beijing, China; ^2^Beijing Institute of Life Science, Chinese Academy of Sciences, Beijing, China

**Keywords:** odorant-binding proteins (OBPs), phase-related behavior, expression profile, locust aggregation, RNAi

## Abstract

Olfactory plasticity, which is one of the major characteristics of density-dependent phase polyphenism, plays critical roles in the large-scale aggregation formation of *Locusta migratoria*. It is still unknown whether odorant-binding proteins (OBPs) are involved in phase-related olfactory plasticity of locusts, despite the confirmed involvement of several types of olfactory perception genes. In this study, we performed a large-scale search for OBPs and verified their expression patterns in the migratory locust. We identified 17 OBPs in the *L. migratoria* genome, of which 10 were novel, and we found their scattering distribution characteristics by mapping the genomic loci. Next, we revealed that these OBPs with close phylogenic relationships displayed similar tissue-specific expression profiles by a combined analysis of qRT-PCR and phylogenetic tree reconstruction. In all identified locust OBPs, seven *OBPs* showed differential mRNA expression levels in antenna tissue between gregarious and solitarious nymphs. Six of these seven OBPs displayed higher mRNA expression in the antennae of gregarious nymphs. The mRNA expression of *LmigOBP2* and *LmigOBP4* increased during gregarization and decreased during solitarization. RNAi experiments confirmed that only *LmigOBP4* regulates the behavioral traits to affect gregarious behavior. These results demonstrated that OBPs also play important roles in the regulation of phase-related behavior of the locusts.

## Introduction

The olfactory sense plays a critical role in behaviors related to food selection, host seeking, courtship, aggregation, and avoidance in insects when receiving external chemical cues (Leal, 2005; Pelosi et al., 2006; Benton, 2007). Despite the diversity of antennal morphs, sensillum types and olfactory gene repertoires among insect species, a general olfactory pathway has been proposed, extending from the reception of odorants to their transmission to odorant receptors (ORs) and activation of an olfactory sensory neuron (OSN) to the projection in the glomerulus in the antennal lobe and coding in higher brain centers (Pelosi et al., 2006; Carey and Carlson, 2011; Leal, 2013). However, the reception of odorants has long been a question because of the complex mixture of numerous and high concentration of olfactory proteins around the dendrites of the OSNs in the sensillum lymph (Pelosi et al., 2006).

As one of the most important chemoreception proteins in insects, odorant-binding proteins (OBPs) have been suggested to play important roles in the reception of odorants. OBPs belong to a large gene family with low protein conservation among family members (Vieira and Rozas, 2011). Generally, these genes are abundantly expressed in chemosensory sensilla, especially in the antennae and labial/maxillary palps. Recent studies supposed that OBPs mainly act as transporters to deliver volatiles or non-volatile chemicals to the ORs and mediate the first step of olfactory signal transmission (Fan et al., 2011). OBPs contribute to insect olfactory perception at various levels. Depending on the types of ligands, OBPs transmit chemical signals to ORs to give rise to corresponding behavioral responses among conspecific insects and across species (Fan et al., 2011; Leal, 2013). OBPs have been reported to be involved in the reception of some oviposition attractants and the determination of reproductive sites by regulating the sensitivity of the insect’s olfactory system (Harada et al., 2008; Pelletier et al., 2010). In addition, OBPs can modulate feeding behavior by regulating the perception to host plant odorants or by affecting sucrose intake in response to bitter compounds (Swarup et al., 2014; Li et al., 2016).

Locusts are one of the most important agricultural pests in the world because of the plague outbreaks resulting from swarm formation and large-scale migration. They display density-dependent behavioral plasticity in transitioning from the disconsolate “solitarious” to the manic “gregarious” phase (Pener and Simpson, 2009). Our recent studies indicated that olfactory regulation related to phase change is a complex process when integrated with the environmental input, gene interaction network, and phenotypic output (Kang et al., 2004; Chen et al., 2010; Guo et al., 2011; Ma et al., 2011). Olfactory perception displays significant differences between solitarious and gregarious locusts in the peripheral and central olfactory nervous systems (Guo et al., 2011; Wang and Kang, 2014; Wang et al., 2015). In the peripheral olfactory perception system, we have found that the olfactory genes, *CSP* and *takeout*, initiate behavioral aggregation by balancing attraction and repulsion responses to conspecific other individuals (Guo et al., 2011). An OR-based signaling pathway mediates the attraction of locusts to aggregation pheromones (Wang et al., 2015). Recent studies have identified 14 OBPs in *Schistocerca gregaria* and determined their distinct sensilla-specific expression patterns (Jiang et al., 2017, 2018). However, it is still unknown whether OBPs are involved in the regulation of phase-related behavioral plasticity in locusts.

In this study, we performed a large-scale search for OBPs in the *Locusta migratoria* genome and analyzed their phylogenetic relationships. A qRT-PCR technique was used to investigate the temporal-spatial expression of OBP genes. RNAi and behavioral assays were used to elucidate the potential function of OBP genes on the behavioral plasticity. We found that OBPs might also be involved in the regulation of the locust phase-related behavior of locusts.

## Materials and Methods

### Insects

The locusts were from the gregarious and solitarious colonies in the Institute of Zoology, CAS, China. Gregarious cultures were reared in large, well-ventilated cages (25 cm × 25 cm × 25 cm) at densities of 200 to 300 insects per cage. Reared solitarious insects were kept in physical, visual, and olfactory isolation that was achieved by ventilating each cage (10 cm × 10 cm × 25 cm) with charcoal-filtered compressed air. Rearing conditions of both colonies were under a 14 h/10 h light/dark photo regime at 30 ± 2°C on a diet of fresh greenhouse-grown wheat seedlings and wheat bran. Fourth-instar gregarious and solitarious nymphs were used in all of the following experiments.

### Experimental Samples

To investigate the tissue-specific expression profiles of OBPs, tissues of antennae, labial palps, brains, wings, and hind legs were collected from gregarious and solitarious nymphs. To investigate the expression profiles of OBPs during phase changes, all the insects were sampled at the same time point (9:00 am) and antenna tissues were collected after 0, 4, 8, and 16 h of solitarization or gregarization. Six individuals were dissected and pooled into one biological replicate and four biological replicates were sampled for each experiment. The sexual ratio of all samples was 1:1. All these samples were stored in liquid nitrogen for further use.

### Identification of OBP Genes and Molecular Cloning of Novel OBPs

We first searched the genes annotated as putative OBPs in the gene set of locust genome (Wang et al., 2014) and the locust transcriptome (Chen et al., 2010). Then, we aligned these sequences and assembled the sequences with high similarity to acquire as long as cDNA sequences by using Geneious Pro 4.8.6 (Biomatters Ltd.). Finally, we confirmed the identified OBPs sequences by Sanger sequencing. According to the assembled sequences, we designed the gene-specific PCR primers for 10 novel OBPs (**Supplementary Table S1**). PCR amplifications were conducted using an ABI veriti thermal cycler and initiated with a 2-min incubation at 94°C, followed by 35 cycles of 94°C, 20 s; 56°C, 20 s; and 72°C, 40 s. PCR products were cloned into T-easy vector (Promega) and sequenced.

### qRT-PCR Analysis

Total RNA was extracted using the RNeasy Mini Kit (QIAGEN) according to the manufacturer’s protocol. The cDNA was reverse-transcribed from 2 μg (0.5 μg from the labial palps) DNase-treated total RNA using MMLV reverse transcriptase (Promega). The mRNA expression level was measured using a SuperReal PreMix Plus (SYBR Green) Kit (Tiangen Biotech, Beijing) and normalized to ribosome protein 49. PCR cycling conditions were based on the manufacturer’s recommendations. PCR amplification was conducted using a Roche Light cycler 480. A melting curve analysis was performed to confirm the specificity of amplification. All samples from IG and CS treatments to test for one OBP gene were run on one individual plate. The qRT-PCR primers of 17 OBP genes are listed in **Supplementary Table S1**.

### Phylogenetic Analysis

MAFFT online version^1^ was used for multiple sequence alignment with the G-INS-1 method and BLOSUM 62 scoring matrix. MEGA 7.0 software was used for the phylogenetic analysis (Kumar et al., 2016). The evolutionary history was inferred using the neighbor-joining method, and the bootstrap consensus tree was inferred from 500 replicates. Branches corresponding to partitions reproduced in less than 50% bootstrap replicates were collapsed. The evolutionary distances were computed using the Poisson correction method, and the units are the number of amino acid substitutions per site.

### RNA Interference

Double-stranded RNA (dsRNA) of green fluorescent protein (GFP), *LmigOBP2* and *LmigOBP4* was prepared using the T7 RiboMAX Express RNAi system (Promega) following the manufacturer’s instructions. Fourth-instar gregarious or solitarious nymphs were injected with 18 μg (6 μg/μl) of dsGFP, dsLmigOBP2, or dsLmigOBP4 in the second ventral segment of the abdomen ∼12 h after molting. The injected gregarious locusts were later marked and placed back into gregarious-rearing cages. Three days later, the effects of RNAi on the mRNA relative expression levels were investigated by qRT-PCR, and the behavior was examined as described below.

### Behavioral Assay

The arena assay experiment was performed in a rectangular Perspex arena (40 cm long × 30 cm wide × 10 cm high) with opaque walls and a clear top. One of the separated chambers (7.5 cm × 30 cm × 10 cm) contained 15 fourth-instar gregarious locusts as a stimulus group, and the other chamber was left empty. Before measurement, the locusts were restricted in a Perspex cylinder for 2 min. Then the locusts were released into the arena and monitored for 300 s. An EthoVision video tracking system (Netherlands, Noldus Information Technology) was used to automatically record individual behavior. A binary logistic model, *P*_greg_ = e^η^/(1+e^η^), η = -2.110 + 0.005 × attraction index + 0.012 × total distance moved + 0.015 × total duration of movement, was used to measure the behavioral phase state of individual locusts (Guo et al., 2011). *P*_greg_ = 1 means fully gregarious behavior and *P*_greg_ = 0 means fully solitarious behavior.

### Data Analysis

Data were analyzed using the IBM SPSS Statistics v.19 software (SPSS Inc., Chicago, IL, United States). Differences between treatments were compared either by Student’s *t*-test or by one-way analysis of variance (ANOVA) followed by a Tukey’s test for multiple comparisons. Behavior-related data were analyzed by Mann–Whitney *U* test because of its non-normal distribution characteristics. Differences were considered significant at *p* < 0.05. Values are reported as means ± SE.

## Results

### Identification, Sequence Alignment, and Genomic Loci of the OBPs

**FIGURE 1:**
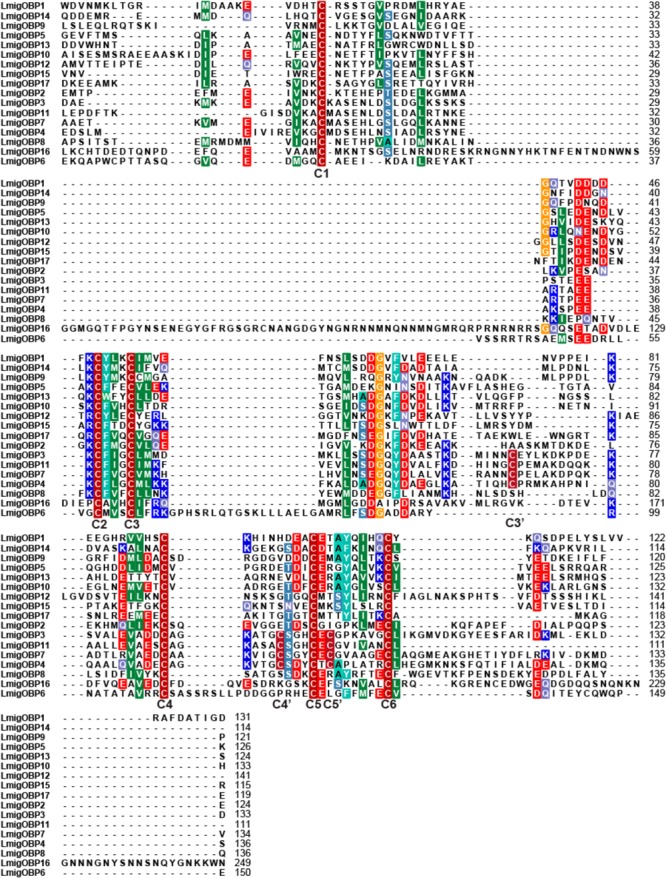
Sequence alignment of 17 *Locusta migratoria* OBPs. Putative signal peptides were removed because of their high substitution rate, and conserved cysteine residues are indicated by capital bold letters (C1–C6 and C3′–C5′).

Based on the *L. migratoria* genome assembly v.2.4, we have identified 17 genes encoding putative OBPs. Of them, seven members that were already known from previous studies: *LmigOBP1, LmigOBP2, LmigOBP3, LmigOBP4, LmigOBP5, LmigOBP15*, and *LmigOBP16* (**Table 1**). Next, we confirmed the cDNA sequences of 10 novel OBPs by PCR cloning and sequencing based on the transcriptomic and genomic sequences (Chen et al., 2010; Wang et al., 2014). The deduced protein lengths of these OBPs ranged from 124 to 271 amino acids, and 10 of the 17 OBPs have predicted signal peptides. Sequence alignment and analysis indicated that these deduced proteins belong to four subtypes: classic, plus-C type-A, plus-C type-B, and atypical OBPs (Jiang et al., 2017). All identified OBPs had six conserved cysteine residues (C1–C6), a three amino acid interval between C2 and C3, and an eight amino acid interval between C5 and C6 (**Figure 1**). Eleven of the 17 OBPs are classic OBPs. *LmigOBP3, LmigOBP4, LmigOBP7*, and *LmigOBP11* had an additional three cysteine residues (C3′, C4′, and C5′) and belong to the plus-C type-A subtype. *LmigOBP6*, which belongs to the plus-C type-B subtype, had two additional cysteine residues with one in front of the C1 residue and one behind the C6 residue. *LmigOBP16* is a long OBP with 271 amino acids and is classified as an atypical OBP.

**Table 1:** Detailed information of 17 locust OBP genes.

Gene Name	Name inGene Set	GenBank No. of Gene	GenBank No. of Protein	Transcript Length	Protein Length	Length of Predicted Signal Peptide	Protein Subtype
LmigOBP1^∗^	LOCMI16968	DQ208934	ABA62340	456	152	21	Classic
LmigOBP2^∗^	LOCMI16967	FJ959361	ACR39388	375	124	0	Classic
LmigOBP3^∗^	LOCMI16976	FJ959365	ACR39392	402	133	0	Plus-C type-A
LmigOBP4^∗^	LOCMI16970	JN247410	AEV45802	465	154	18	Plus-C type-A
LmigOBP5^∗^	LOCMI16973	JQ766964	AFL03411	653	151	25	Classic
LmigOBP6	LOCMI16975	JN129989	AEX33162	595	166	16	Plus-C type-B
LmigOBP7	LOCMI16974	JN129990	AEX33163	419	134	0	Plus-C type-A
LmigOBP8	LOCMI16965	JN129991	AEX33164	497	157	21	Classic
LmigOBP9	LOCMI16978	JN129992	AEX33165	477	145	24	Classic
LmigOBP10	LOCMI16966	JN129993	AEX33166	462	133	0	Classic
LmigOBP11	LOCMI16971	JN129994	AEX33167	410	134	23	Plus-C type-A
LmigOBP12	LOCMI17552	JN129995	AEX33168	537	159	18	Classic
LmigOBP13	LOCMI16977	JN129987	AEX33160	526	158	34	Classic
LmigOBP14	LOCMI16969	JN129988	AEX33161	450	138	25	Classic
LmigOBP15^∗^	LOCMI17559	KU865299	AMO66404	417	138	23	Classic
LmigOBP16^∗^	LOCMI17553	KU865300	AMO66405	877	271	22	Atypical
LmigOBP17	LOCMI16972	MH176616	–	693	138	19	Classic

*OBPs identified in previous studies are marked with an^∗^.*

These 17 OBPs genes were scattered on 16 scaffolds of the whole genome sequences (**Table 2**). Only *LmigOBP5* and *LmigOBP8* were on the same scaffold with an ∼32 kb intergenic region. The length of the OBP genes ranged from 10374 bp (*LmigOBP2*) to 79617 bp (*LmigOBP9*), in which *LmigOBP1, LmigOBP6, LmigOBP8, LmigOBP9*, and *LmigOBP16* had seven exons and the other 12 OBPs all had six exons. The length of the exons and introns ranged from 20 bp to 210 bp and 83 bp to 58448 bp, respectively. Like the other coding genes of *L. migratoria* (Wang et al., 2014), the OBP genes also had many long introns and the lengths of ∼55% of the introns is more than 5 kb (**Table 2**).

**Table 2:** Genomic loci of OBP genes in the *L. migratoria* genome.

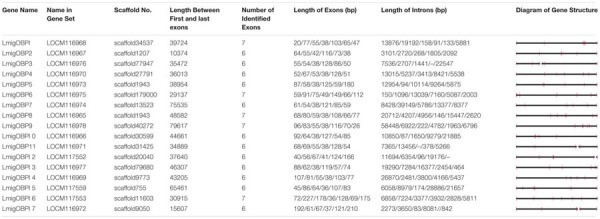

### Tissue-Specific Expression Profiles and Phylogenetic Analysis of the OBPs

**FIGURE 2:**
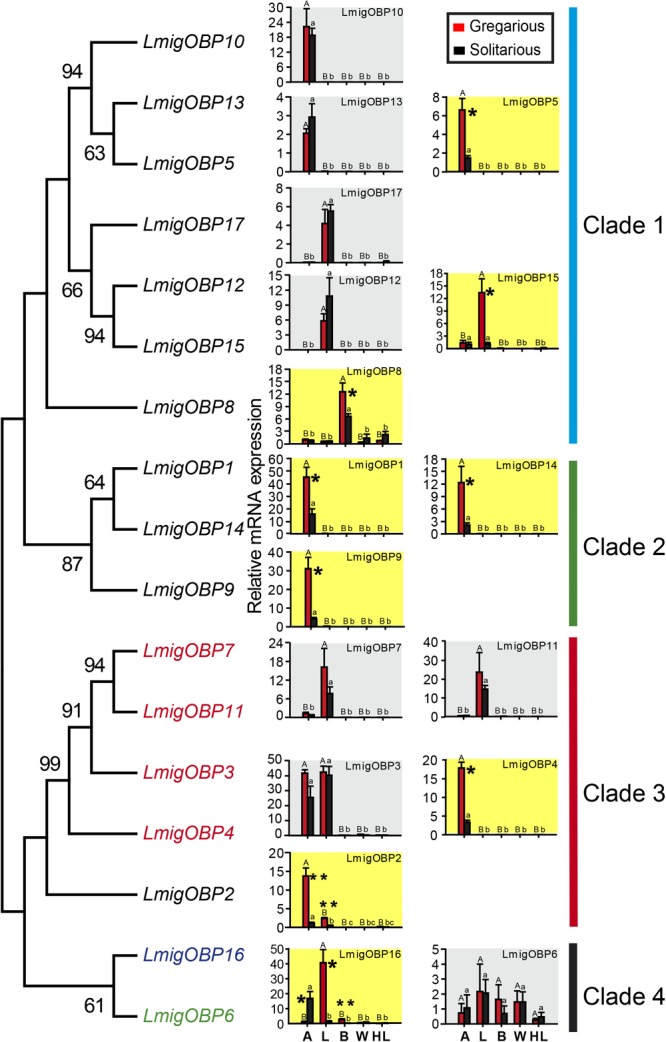
Phylogeny of locust OBPs and their tissue expression profiles in gregarious and solitarious fourth-instar nymphs. Consensus unrooted trees were generated with 500 bootstrap trials using the neighbor-joining method and presented with a cutoff value of 50. A, antenna; L, labial palp; B, brain; W, wing; HL, hind leg. For tissue expression profiles analysis, one-way ANOVA followed by Tukey’s test for multiple comparisons was used. Means labeled with the same letter (capital letters for gregarious and lower-case letters for solitarious) within each treatment are not significantly different. For comparison between gregarious and solitarious, Student’s *t*-test was used and ^∗∗^ means *p* < 0.01 and ^∗^ means *p* < 0.05. Differences were considered significant at *p* < 0.05. Values are reported as means ± SE. Taxon names in black, classic OBPs; in red, plus-C type-A OBPs; in green, plus-C type-B OBPs; in blue, atypical OBPs.

We determined the expression levels of locust OBP genes in five tissues including the antenna, labial palp, brain, wing, and hind leg. Their expression profiles can be divided into five patterns: (a) Eight OBPs are antenna-rich expressions, including *LmigOBP1, LmigOBP2, LmigOBP4, LmigOBP5, LmigOBP9, LmigOBP10, LmigOBP13*, and *LmigOBP14*; (b) Five OBPs are labial palp-rich expressions, including *LmigOBP7, LmigOBP11, LmigOBP12, LmigOBP15*, and *LmigOBP17*; (c) Two OBPs are antenna and labial palp-rich expressions, including *LmigOBP3* and *LmigOBP16*; (d) One OBP is a brain-rich expression, including *LmigOBP8*; and (e) One OBP is a multi-tissue expression, including *LmigOBP6* (**Figure 2**).

We further compared the expression levels of these OBPs between gregarious and solitarious locusts. Nine of 17 OBP genes were differentially expressed in a range of tissues between the gregarious and solitarious locusts (**Figure 2**; shading in yellow). Seven OBPs, *LmigOBP1, LmigOBP2, LmigOBP4, LmigOBP5, LmigOBP9, LmigOBP14*, and *LmigOBP16*, were differentially expressed in the antennal tissue (Student’s *t*-test, *t* = 3.311, 4.973, 7.747, 3.510, 3.689, 3.079, 3.411; *p* = 0.002, 0.004, 0.001, 0.017, 0.014, 0.027, 0.041, respectively). Except for *LmigOBP16*, the other six OBPs were highly expressed in the antennal tissue of gregarious locusts. Three OBPs, *LmiOBP2, LmigOBP15*, and *LmigOBP16*, were highly expressed in the labial palp tissue of gregarious locusts (Student’s *t*-test, *t* = 8.869, 3.704, 4.493; *p* = 0.002, 0.033, 0.020, respectively). Two OBPs, *LmigOBP8* and *LmigOBP16*, were highly expressed in the brain tissue of gregarious locusts (Student’s *t*-test, *t* = 2.639, 4.074, *p* = 0.039, 0.007, respectively).

The 17 OBPs were classified into four clades according to their phylogenetic relationships. Except for *LmigOBP2* in clade 3, the classic OBPs are all in clades 1 and 2. Four plus-C type-A OBPs are all in clade 3. *LmigOBP6* (plus-C type-B OBP) and *LmigOBP16* (atypical OBP) are both in clade 4 (**Figure 2**). In clade 1, seven OBPs are divided into three branches, which represent antenna-, labial palp-, and brain-rich expression OBPs. OBPs with close phylogenetic relationships had similar gene expression patterns, such as the gene expression among *LmigOBP5, LmigOBP10*, and *LmigOBP13*. In clade 2, three OBPs had a similar tissue expression pattern and were all highly expressed in gregarious antenna tissue. In clade 3, four OBPs displayed diverse antenna-labial palp expression patterns. The *LmigOBP2* gene was expressed significantly higher in both antenna and labial palp tissues of gregarious nymphs (**Figure 2**). In clade 4, *LmigOBP16* was significantly highly expressed in the solitarious antenna, gregarious labial palp, and brain. The expression level of *LmigOBP6* displayed no significant differences in different tissues or phase individuals (**Figure 2**).

### Phylogenetic Analysis of Related Insect OBPs

**FIGURE 3:**
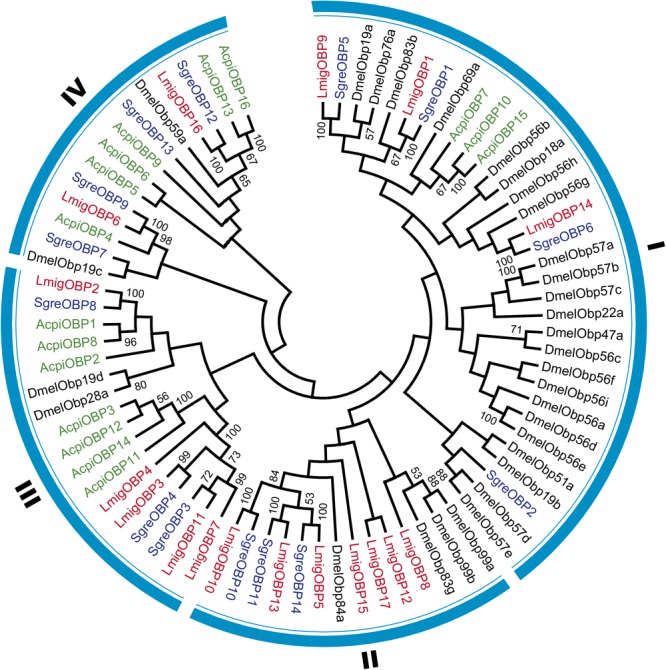
Phylogenetic analysis of OBPs in four representative insect species. Consensus unrooted trees were generated with 500 bootstrap trials using the neighbor-joining method and presented with a cutoff value of 50. Taxon names in red, *Locusta migratoria*; in black, *Drosophila melanogaster*; in blue, *Schistocerca gregaria*; in green, *Acyrthosiphon pisum*.

We used 31 OBPs from holometabolous *Drosophila melanogaster* and 14, 17, and 16 OBPs from hemimetabolous *S. gregaria, L. migratoria*, and *Acyrthosiphon pisum*, respectively, for phylogenetic analysis (**Figure 3** and **Supplementary Data Sheet S1**). The phylogenetic relationships indicated that *L. migratoria* OBPs is distributed in four families together with the OBPs of other insect species. Family I includes most OBPs from *D. melanogaster* and many OBPs are specific to *D. melanogaster*. *SgreOBP2* has no homolog in *L. migratoria*, and *AcpiOBP10* and *AcpiOBP15* are *A. pisum*-specific OBPs. In family II, most OBPs are from *L. migratoria* and no *A. pisum* OBPs distribute in this family. *LmigOBP12* and *LmigOBP15*, which are highly expressed in labial palp tissue, have no homologs in other insect species. In family III and IV, most OBPs are from hemimetabolous insect species and 13 of 16 *A. pisum* OBPs distribute in these two families.

### Time-Course Gene Expression Profiles During Locust Phase Change

**FIGURE 4:**
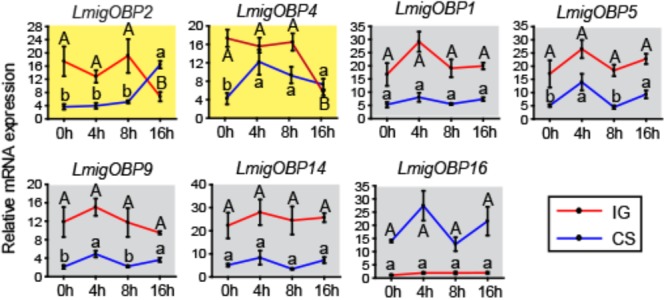
Time-course expression profiles of OBPs showing differential expressions in antennae between gregarious and solitarious fourth-instar nymphs. IG, isolation of gregarious nymphs; CS, crowding of solitarious nymphs. Shading in yellow, differentially expressed in both IG and CS processes; shading in gray, no different expression in either the IG or CS process. One-way analysis of variance (ANOVA) followed by Tukey’s test for multiple comparisons was used. Means labeled with the same letter (capital letters for IG process and lower-case letters for CS process) within each treatment are not significantly different. Differences were considered significant at *p* < 0.05. Values are reported as means ± SE.

To determine the relationships between OBPs and phase change in the locusts, we investigated the time-course expression profiles of seven differentially expressed OBPs in antenna tissue (**Figure 4**). *LmigOBP2* or *LmigOBP4* displayed a reverse expression pattern after IG (isolation of gregarious locusts) or CS (crowding of solitarious locusts). The expression level of *LmigOBP2* did not change until 16 h during the IG or CS process (ANOVA, *F*_3,12_ = 6.726, 53.345; *p* = 0.007, 0.000, respectively). The expression of *LmigOBP4* decreased significantly after IG for 16 h, increased rapidly from 0 to 4 h, and then stayed at a stable level during the CS process (ANOVA, *F*_3,12_ = 7.092, 21.549; *p* = 0.005, 0.000, respectively). Although there are small changes of *LmigOBP5* and *LmigOBP9* expression because of smaller variation in those trials during CS time-course, the expression of *LmigOBP1, LmigOBP5, LmigOBP9, LmigOBP14*, and *LmigOBP16* might be considered to have no changes during IG and CS time-courses because most of the expression changes of OBPs were not significant (IG, ANOVA, *F*_3,12_ = 2.585, 1.590, 0.719, 0.225, 1.215; *p* = 0.102, 0.243, 0.561, 0.877, 0.350, respectively. CS, ANOVA, *F*_3,12_ = 2.680, 6.083, 7.414, 3.017, 1.463; *p* = 0.094, 0.009, 0.005, 0.053, 0.278, respectively). So, we categorized these five OBPs into one pattern that no response to IG and CS treatments. Therefore, *LmigOBP2* and *LmigOBP4* would be related to the phase changes of the locusts because their expression responds to the time-course treatments (IG and CS).

### Effects of LmigOBP2 and LmigOBP4 RNAi on Phase-Related Behavior

**FIGURE 5:**
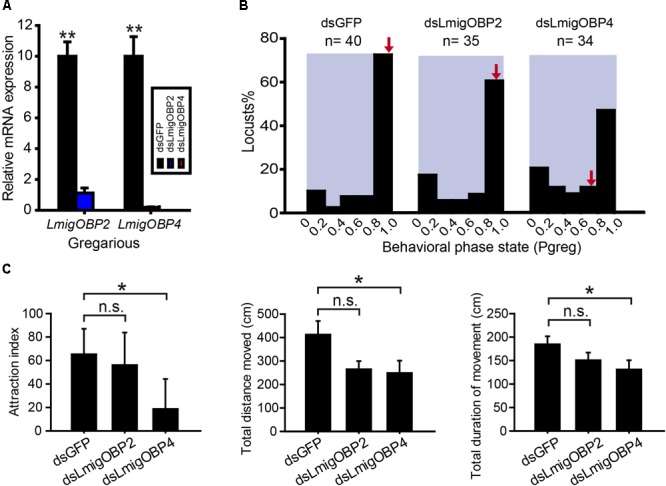
Effects of RNAi knockdown of *LmigOBP2* and *LmigOBP4* genes in their expression levels and behavioral phenotypes in gregarious locusts. **(A)** Relative mRNA expressions of *LmigOBP2* and *LmigOBP4* in antennal tissue after dsLmigOBP2 or dsLmigOBP4 injection. ^∗∗^*p* < 0.01. **(B)** Effect of dsGFP, dsLmigOBP2, or dsLmigOBP4 injection on the behavioral phase state in fourth-instar nymphs. Arrows indicate median *P*_greg_ values. *n* = number of individuals. **(C)** Effects of dsLmigOBP2 or dsLmigOBP4 injection on attraction index, total distance moved, and total duration of movement. ^∗^*p* < 0.05; n.s., not significant.

To investigate the potential functional significance of *LmigOBP2* and *LmigOBP4*, RNAi and behavioral assays were performed to identify their functions *in vivo*. We injected dsRNAs to knock down their expression levels in gregarious and solitarious nymphs, respectively. In gregarious locusts, compared with the double-stranded GFP-injected (dsGFP) control, the expressions of both genes, *LmigOBP2* and *LmigOBP4*, decreased significantly after the injections of double-stranded *LmigOBP2* or *LmigOBP4* (dsLmigOBP2 or dsLmigOBP4) (Student’s *t*-test, *t* = 9.043, 8.295; *p* = 0.001, 0.014, respectively; **Figure 5A**). Because of the low identity (18.78%) between dsLmigOBP2 and dsLmigOBP4 fragments, it is quite low for the possibility that *LmigOBP2* is knocked down by dsLmigOBP4 injection, and *LmigOBP4* is knocked down by dsLmigOPB2 injection (**Supplementary Figure S1**). A behavioral assay indicated that the behavioral traits were significantly altered after *LmigOBP4 gene* knockdown (Mann–Whitney *U* = 471, *p* = 0.023) and median *P*_greg_ changed from 0.995 to 0.785 (**Figure 5B**). Correspondingly, the attraction index, total distance moved and total duration of movement in dsLmigOBP4-injected locusts were significantly reduced to 28.7, 60.3, and 70.9% of the dsGFP-injected locusts (Mann–Whitney *U* = 486, 461, 492.5; *p* = 0.035, 0.018, 0.042, respectively; **Figure 5C**). However, *LmigOBP2* knockdown didn’t change the behavioral phase state (Mann–Whitney *U* = 564, *p* = 0.149; **Figure 5B**) and median ˆ*P*_greg_ changed a little from 0.995 to 0.980 (**Figure 5B**). The attraction index, total distance moved and total duration of movement in dsLmigOBP2-injected locusts were not altered at all (Mann–Whitney *U* = 644.5, 589, 541.5; *p* = 0.556, 0.239, 0.092, respectively; **Figure 5C**). In solitarious locusts, compared with the double-stranded GFP-injected (dsGFP) control, the expressions of both genes, *LmigOBP2* and *LmigOBP4*, decreased significantly after the injections dsLmigOBP2 or dsLmigOBP4 (Student’s *t*-test, *t* = 14.548, 6.837; *p* = 0.000, 0.021, respectively; **Supplementary Figure S2A**). The behavioral phase state was not affected after *LmigOBP2* or *LmigOBP4* knockdown (Mann–Whitney *U* = 305, 312, *p* = 0.706, 0.992, respectively; **Supplementary Figure S2B**). The attraction index, total distance moved, and total duration of movement were not changed at all after *LmigOBP2* knockdown (Mann–Whitney *U* = 319, 316, 315.5; *p* = 0.904, 0.865, 0.815, respectively; **Supplementary Figure S2C**).or *LmigOBP4* knockdown (Mann–Whitney *U* = 306.5, 283, 278; *p* = 0.900, 0.567, 0.318, respectively. **Supplementary Figure S2C**).

## Discussion

This study describes the identification, temporal-spatial expression, and effects on phase-related behavior of OBPs in the migratory locust. Ten OBPs were identified as novel member of OBPs in the locusts. OBPs with close phylogenetic relationships displayed similar tissue-specific expression patterns. Through filtering genes related to the time-course of phase changes in antenna tissue, we suggested that *LmigOBP4* might be involved in the regulation of the behavioral transition of in locusts.

In this study, we identified 17 OBPs in the *L. migratoria* genome. The number of *L. migratoria* OBPs is more than that of the other three orthopteran species, *S. gregaria* (14 OBPs), *Oedaleus asiaticus* (15 OBPs), and *Ceracris kiangsu* (7 OBPs). Most *L.migratoria* OBPs have high homology with those of *S. gregaria*. We did not find the orthologs of *SgreOBP2, SgreOBP7*, and *SgreOBP13* in *L. migratoria*. Whereas several *L. migratoria* OBPs including *LmigOBP4, LmigOBP8, LmigOBP12, LmigOBP15*, and *LmigOBP17* have no orthologs in *S. gregaria* (Jiang et al., 2017). The numbers of OBPs differed markedly among insect species and ranged from 4 (*Pediculus humanus*) to 81 (*Anopheles gambiae*) in the genome (Vieira and Rozas, 2011). Compared to species of Diptera, Lepidoptera, and Coleoptera, orthopteran species have less expansion of the OBP family, which is similar to several representative hemipteran and hymenopteran insects (Fan et al., 2011; Vieira and Rozas, 2011; He and He, 2014; Jiang et al., 2017). However, *L. migratoria* has a large expansion of the OR family (142 ORs) (Wang et al., 2015). Considering the transport of odorant molecules from OBPs to ORs, it is probable that one OBP might transport multiple odors to variant ORs with different binding capabilities (Leal, 2013).

We revealed that most locust OBPs with a close phylogenetic relationship have similar tissue-specific expression profiles (**Figure 2**). In general, genes with similar tissue-specific expression patterns could be regulated simultaneously to perform related functions, especially for members of a gene family (Stevens et al., 2008; Huang et al., 2015; Gu, 2016). This phenomenon also suggests that phylogenetically correlated OBPs probably constitute a functional cluster to separate and discriminate odors in a complex environmental context. However, the knowledge of gene expression patterns *per se* is insufficient to infer gene function (Yanai et al., 2006). Functional confirmation of these OBPs needs further analysis of their protein distributions on a range of sensilla, protein structures, ligand-binding properties, and behavioral phenotypes (Harada et al., 2008; Yu et al., 2009; Li et al., 2016).

Most OBPs were highly expressed in antennal or labial palp tissues, indicating that locust OBPs might mainly be involved in olfactory or gustatory functions, as are those of other insect species (Fan et al., 2011; Swarup et al., 2011). Increasing evidence indicates that OBPs are also extensively expressed in variant tissues, such as the brain, maxillary galeae, mandibular glands, and legs (Forêt and Maleszka, 2006; Gu et al., 2011; Iovinella et al., 2011; Yoshizawa et al., 2011). Interestingly, *LmigOBP8* is highly expressed in brain tissues of both gregarious and solitarious nymphs. In honey bees, the *OBP10* gene begins to express in the pupae and increases to the highest level in the brain of newly emerged bees (Forêt and Maleszka, 2006). As carriers of small molecules, OBPs might also transport some ligands for neural development or signal transmission. In the locusts, some ORs and IRs are also detected in the brain tissue (Wang et al., 2015). Whether *LmigOBP8* is involved in ligand transport to the ORs and IRs needs further investigation. The significantly different expression of *LmigOBP8* between the two phases suggested its potential function in the regulation of phenotypic plasticity in the central nervous system. In addition, in *Bombyx mori*, the expression of OBPs and CSPs in the female pheromone glands suggested their function in the solubilization and delivery of pheromonal components (Dani et al., 2011). In *D. melanogaster*, Obp57d and Obp57e were co-expressed in the taste sensilla on the legs to sense host plant toxins (Harada et al., 2008; Yasukawa et al., 2010). The evidences indicated that OBPs could be involved in multiple physiological processes besides olfactory perceptions.

Olfaction plays critical roles in tuning behavior to the rapid adaptation to environmental change in the locust, especially changes of population density (Guo et al., 2011; Wang et al., 2015). Here, we identified that an OBP, *LmigOBP4*, was involved in the phase-related behavior of locusts. The orthologs of several OBP members of *L. migratoria* in family III, which *LmigOBP4* belongs to, have recently been reported to distribute in the sensilla chaetica of the antennae in *S. gregaria* (Jiang et al., 2017, 2018). So, we inferred that *LmigOBP4* might have similar sensilla distribution in the antenna. The sensilla chaetica can not only perceive stimulation resulting from contact chemical molecules (Isidoro et al., 1998), but also volatiles (Ma et al., 2018). Several body volatiles or cuticular hydrocarbons, probably acting as pheromones, were involved in the induction of phase-related behavior (Heifetz et al., 1997; Wei et al., 2017). Therefore, *LmigOBP4* might bind with these volatiles and transmit the chemical cues to activate OSNs. Differential expression of *LmigOBP4* between two phases might contribute to differential olfactory sensitivity and inspire different behavioral responses to conspecific volatiles. The knockdown of *LmigOBP2*, which also displayed differential expression during phase changes, did not change the behavioral traits in our arena assay. The possible reason is that *LmigOBP2* might contribute to differential sensitivity in response to contact chemical compounds or plant volatiles. Our previous studies indicated that two olfactory genes, *LmigCSP3* and *LmigTO1*, can regulate the attractive/repulsive responses to conspecifics during locust phase changes (Guo et al., 2011). So, these olfactory proteins might play different roles in chemical perception for a rapid adjustment or long-term adaptation during aggregation.

OBPs bridge the interaction between odorants and ORs (Xu et al., 2005). The identification of tissue-specific OBPs expression patterns provides cues for research about their functions in variant tissues of the locusts. The functional confirmation of *LmigOBP4* in locust phase-related behaviors will benefit further studies of the interactions between odorants and ORs. These findings provide further insights into olfactory plasticity in related insect species.

## Author Contributions

LK, WG, and XW conceived the study. WG, DR, LZ, and JS conducted the experiments. WG, LK, FJ, and XW interpreted the results. WG drafted the preliminary manuscript. LK and XW refined and approved the final manuscript.

## Conflict of Interest Statement

The authors declare that the research was conducted in the absence of any commercial or financial relationships that could be construed as a potential conflict of interest.

## Footnotes

1 https://mafft.cbrc.jp/alignment/server/

## Supplementary Material

FIGURE S1 Sequence alignment of cDNA fragments that were used for dsRNA synthesis of *LmigOBP2* and *LmigOBP4*.

FIGURE S2 Effects of RNAi knockdown of *LmigOBP2* and *LmigOBP4* genes in their expression levels and behavioral phenotypes in solitarious locusts. **(A)** Relative mRNA expressions of *LmigOBP2* and *LmigOBP4* in antennal tissue after dsLmigOBP2 or dsLmigOBP4 injection. ^∗∗^*p* < 0.01. **(B)** Effect of dsGFP, dsLmigOBP2 or dsLmigOBP4 injection on the behavioral phase state in fourth-instar nymphs. Arrows indicate median *P*_greg_ values. *n* = number of individuals. **(C)** Effects of dsLmigOBP2 or dsLmigOBP4 injection on attraction index, total distance moved and total duration of movement. n.s., not significant.

TABLE S1 Primers for OBPs sequence cloning, qRT-PCR and RNAi.
