# Supplementary figures and images for: Identification of Odorant-Binding Proteins (OBPs) and Functional Analysis of Phase-Related OBPs in the Migratory Locust
Source: Front Physiol. 2018 Jul 20;9:984. doi: 10.3389/fphys.2018.00984 (PMC6062766; doi:10.3389/fphys.2018.00984)

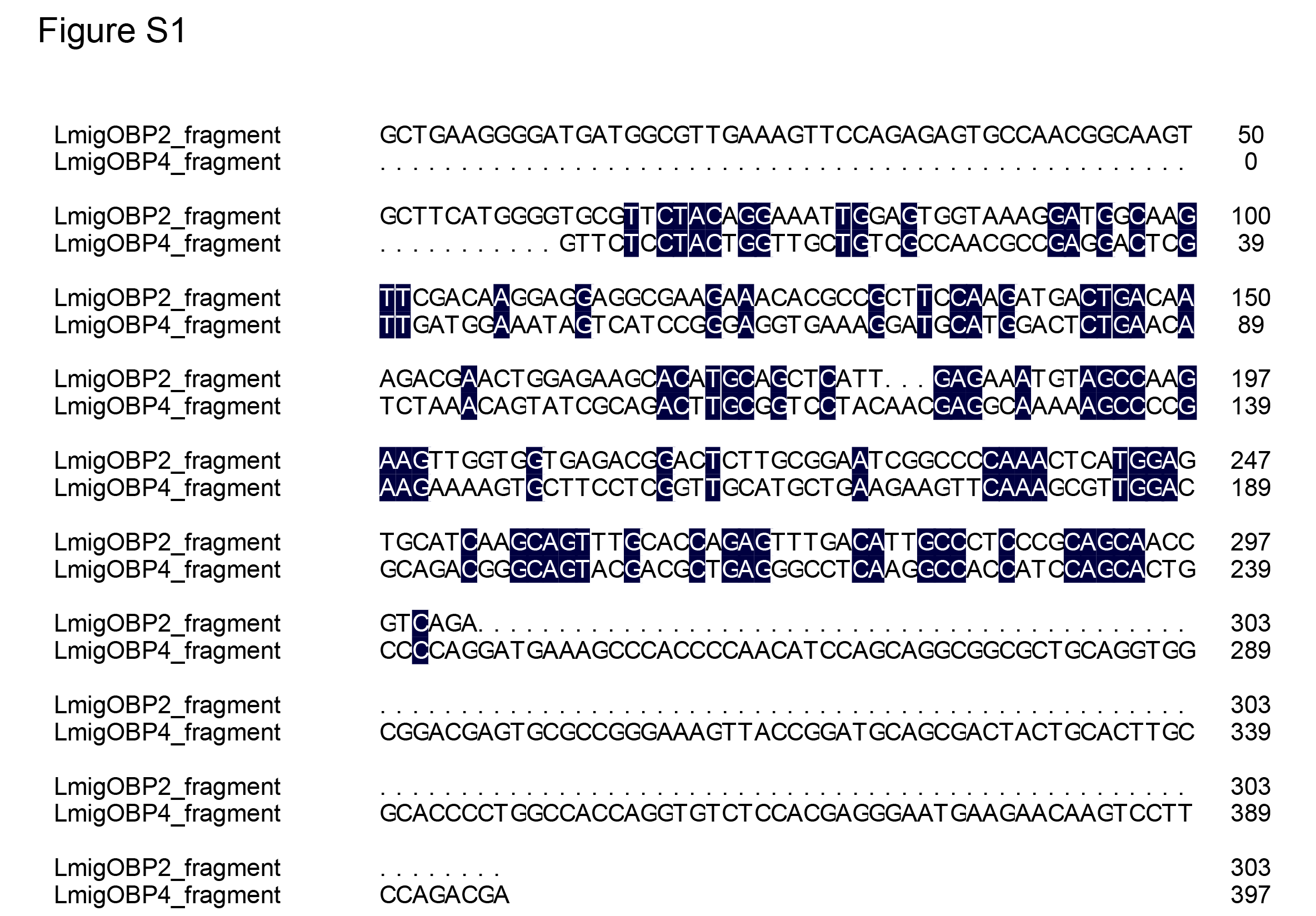

Supplement: FIGURE S1 — Sequence alignment of cDNA fragments that were used for dsRNA synthesis of LmigOBP2 and LmigOBP4. [file Image_1.TIF]

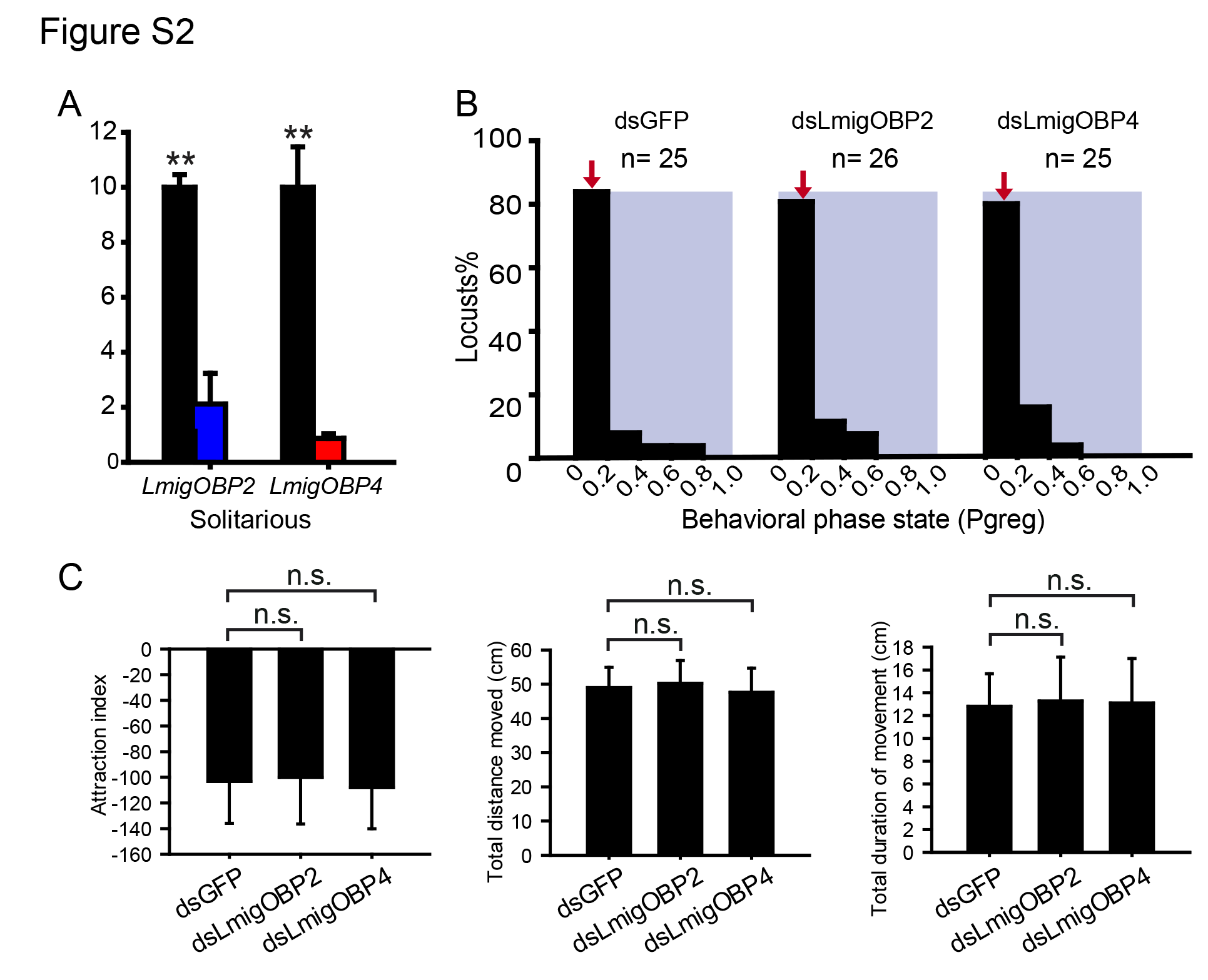

Supplement: FIGURE S2 — Effects of RNAi knockdown of LmigOBP2 and LmigOBP4 genes in their expression levels and behavioral phenotypes in solitarious locusts. (A) Relative mRNA expressions of LmigOBP2 and LmigOBP4 in antennal tissue after dsLmigOBP2 or dsLmigOBP4 injection. ∗∗p < 0.01. (B) Effect of dsGFP, dsLmigOBP2 or dsLmigOBP4 injection on the behavioral phase state in fourth-instar nymphs. Arrows indicate median Pgreg values. n = number of individuals. (C) Effects of dsLmigOBP2 or dsLmigOBP4 injection on attraction index, total distance moved and total duration of movement. n.s., not significant. [file Image_2.TIF]
