# Supplementary material for: Identification of Odorant-Binding Proteins (OBPs) and Functional Analysis of Phase-Related OBPs in the Migratory Locust
Source: Front Physiol. 2018 Jul 20;9:984. doi: 10.3389/fphys.2018.00984 (PMC6062766; doi:10.3389/fphys.2018.00984)
Supplement: TABLE S1 — Primers for OBPs sequence cloning, qRT-PCR and RNAi. [file Table_1.PDF]

Table S1. Primers for OBPs sequence cloning, qRT-PCR and RNAi.

	Gene name	Forward primer	Reverse primer
OBPs sequence cloning	*LmigOBP6*	GCAGCTCCTCCTCGTCTCC	GCAATGCAGTGCAGGTTAGTG
	LmigOBP7	GCGGCTGAGACCAAAGTAA	GTCTTGATCTAAACGTCCATCTT
	LmigOBP8	CTGCTGTACGACTGCTGCTT	GAAACTACGCCTGCCTCACT
	LmigOBP9	GCACAGACGCCACCGCCAC	CATCCACATCAAGGGAATAAGAA
	LmigOBP10	CAGCGATCTCGGAGTCAAT	GGAGTTCCTCTGGTGCTTTGT
	LmigOBP11	CCGTCGCCACCATGAGGT	GCAAGCAGTTGGCAACGATG
	LmigOBP12	TGGCGGTCATCCTCACTG	GCTTGCTGAATTATTTATTACTC
	LmigOBP13	CTGCGACCCCAGACACGA	TTGTTCAGTGCAGGACTACAAA
	LmigOBP14	GTGTAGCGTCGACGGCCGGCA	ATGGCATCTCAATGTACTTCAG
	LmigOBP17	TCGCAGATAAACATGAAAGCCT	GCAGCAATCCGTTCCATAGTG
qRT-PCR	*LmigOBP1*	AAGGAAGAAGGCCATAGGGTT	TGCACGAACTACCAGGCTGTA
	LmigOBP2	GACAAGGAGGAGGCGAAGAAA	CAAGAGTCCGTCTCACCACCAA
	LmigOBP3	ATTGTTGTCTTCAGATGGGCAGTA	TCTCGTCGGGTTTGTCCTTG
	LmigOBP4	CAGACTTGCGGTCCTACAACGA	GTGCTGGATGGTGGCCTTGA
	LmigOBP5	TCGAGTGCGTGCTGGAGAA	CCTTCGTGGCGAGGAATACAG
	LmigOBP6	ATACTCAGGGAATACGCCAAGACT	CTCACCATGCACCCAACCAG
	LmigOBP7	CATCTGGGTAGCCTGGGTC	AGGGAGTCCGCCTTCTTCT
	LmigOBP8	AGTGGATGGACGACGAAGGC	TCCGATGATCCTGTCGCAGA
	LmigOBP9	GTCGAAGGAATCCAGGAAGG	GTCCAGCATGTCGATGAACC
	LmigOBP10	TAAGTGCTTCATACACTGCCTGAC	TAGGGAAACGGCGAGTCATC
	LmigOBP11	CAGAACCAACAAGGAGGCG	TCGGCAACTTCTAACAAAGC
	LmigOBP12	CTCGGTGTTACCTGGAATGT	GTCCCAGAAGACTTGGAATT
	LmigOBP13	ACGTATGGCACAACACCGACATCC	ATCCCGTCTTATCCAGCAAG
	LmigOBP14	CAGATGACGGAGTATTTGATGC	TAGTCCTTTGGTGCCTGCTTGA
	LmigOBP15	GAACGATTCAGTGGCGAGATGT	AGTTGAGACTTCCGTCCGATGT
	LmigOBP16	TTTCGTGGGAGCGGTAGATGTA	GCCTCTGCCTCATTCCATTCAT
	LmigOBP17	TCGCAGATAAACATGAAAGCCT	GCAGCAATCCGTTCCATAGTG
RNAi	*LmigOBP2*	GCTGAAGGGGATGATGGCG	TCTGACGGTTGCTGCGGGA
	LmigOBP4	GTTCTCCTACTGGTTGCTGTC	TCGTCTGGAAGGACTTGTTCT
