# Supplementary material for: Identification of Odorant-Binding Proteins (OBPs) and Functional Analysis of Phase-Related OBPs in the Migratory Locust
Source: Front Physiol. 2018 Jul 20;9:984. doi: 10.3389/fphys.2018.00984 (PMC6062766; doi:10.3389/fphys.2018.00984)
Supplement: Supplementary file 4 [file Data_Sheet_1.DOCX]

>LmigOBP1

WDVNMKLTGRIMDAAKEVDHTCRSSTGVPRDMLHRYAEGQTVDDDDFKCYLKCIMVEFNSLSDDGVFVLEEELENVPPEIKEEGHRVVHSCKHINHDEACETAYQIHQCYKQSDPELYSLVVRAFDATIGD

>LmigOBP2

EMTPEFMEIVNKCKTEHEPTEDELKGMMALKVPESANGKCFMGCVLQEIGVVKDGKFDKEEAKKHAASKMTDKDELEKHMQLIEKCSQEVGGETDSCGIGPKLMECIKQFAPEFDIALPQQPSE

>LmigOBP3

DAEKMKEAVDKCKASENLDSLDGLKSSKSPSTEEEKCFIGCLMMDMKLLSSDGQYDAASTKDMINNCEYLKDKPDEKSVALEVADDCAGKATGCSGHCECGPKAVGCLIKGMVDKGYEESFARIDKMLEKLDD

>LmigOBP4

EDSLMEIVIREVKGCMDSEHLNSIADLRSYNEAKSPEEKCFLGCMLKKFKALDADGQYDAEGLKATIQHCPRMKAHPNIQQAALQVADECAGKVTGCSDYCTCAPLATRCLHEGMKNKSFQTIFIALDEALDKMQS

>LmigOBP5

GEVFTMSQLKAAVNECNDTYFLSQKNWDTVFTTGSLEDENDLVAKCFFECVLEKTGAMDEKGNINSDITKAVFLASHEGTGTAVQGHDDLIDMCVPGRDETDICERGYALVKCVTVEELSRRQARK

>LmigOBP6

EKQAPWCPTTASQGVQEDMGQCAEEIKDAILREYAKTVSSRRTRSAEMSEEDRLLVGCMVSCLFRKGPHSRLQTGSKLLLAELGAMRLFSDGADDARYRNATATAVRRCSASSRSLLPDDGGPRHECELGFFMFECVSDQITEYCQWQPE

>LmigOBP7

AAETKVMEGIKACMASEHLGSLGQLKANNEARTPEEKCFVGCVMKHLHVLNSEGQYDLALVKERANNCPELAKDPQKKADTLRVAEDCAAKVIGCSGYCECGVAAGECLAQGMEAKGHETIYDFLRKIVDKMDV

>LmigOBP8

APSITSTEMRMDMMVIQHCNETHPVALIDMNKALINKKIEPQNTVFKCFVFCLLNKYEWMDDEGGFLIANMKHNLSDSHLDQLSIDFIVYKCSATGSSDKCERAYRFTECFWGEVTKFPENSDEKYEDPDLFALYQ

>LmigOBP9

LSLEQLRQTSKIVRNMCLKKTGVDLALVEGIQEGQFPDNQDLKCYMKCCMGAMQVLRQGRYNVNAAKNQADKMLPPDLKGRFIDMLDACSDRGDGVDDDCEMAYQLTKCSYETDKEIFLFP

>LmigOBP10

AISESMSRAEEAASKIDIPELFEECNETFTIPKVTLNYFFSHGRLQNENDYGSKCFVHCLTDRSGEIDSDGNFDVDLIKVMTRRFPNETNIEGLNEMVETCVADRGETDFCERAYGLVSCLVKEKLARLGNSH

>LmigOBP11

LEPDFTKGISDVKACMASENLDSLDALRTNKEARTAEEKCFIGCIMKFVEVLNSDGQYDVALFKDHINGCPEMAKDQQKKAALLEVAESCAGKASACSGHCECGVIVANCL

>LmigOBP12

AMVTTEIPTEDILQRVQVCNKTYPVSQEMLRSLASTGGLLSDESDVNTRCYLECYERLGGTVNKDGKFNPEKAVTLLVSYYPKIAELGVDSVTEILKNCNSKSGTGQCMTSYLIRNCFIAGLNAKSPHTSVFDTSSSHIKL

>LmigOBP13

DDVWHNTDIPATMAECNATFRLGWRCWDNLLSDGHVIDESKYQQKCWFYCLLDETGSMHADGAFDKDLLKTVLQGFPNGSSLAHLDETTYTCVAQRNEVDLCERAYAVVKCIMTEELSRMHQSS

>LmigOBP14

QDDEMREMMDQLHQTCVGESGVSEGNIDAARKGNFIDDGNLKCYMKCIFVQMTCMSDDGVFDADTAIAMLPDNLKDVASKALNACKGEKGSDACDTAFKINQCLFKQAPKVRIL

>LmigOBP15

VNVDIETIWRECNETFPASEEALISFGKNGTIPDENDSVARCFTDCYGKKTTLLTSDGSLNWTTLDFLMRSYDMKPTAKETFGKCQKNTSNVECMKSYLSLRCVAETVESLTDIR

>LmigOBP16

LKCHTDEDTQNPDEFQEVAAMCMKNTSGSELNRNDRESKRNGNNYHKTNFENTNDNWNSGGMGQTFPGYNSENEGYGFRGSGRCNANGDGYNGNRNNMNQNNMNGMRQRPRNRNRRSGQQSETADVDLEDIEPCAVHCIFRQMGMLGDDAIPDRSAVAKVMLRGVKDTEVKDFVQEAVEDCFDQVESDRKGSKCEFSKNVALCLRQKGRENCEDWGEQDGDQQSNQNKNGNNNGNYSNNSNQYGNKKWN

>LmigOBP17

DKEEAMKILRASVDKCSAGYGLSRETTQYIVRHNFTIKDENDENQRCFVQCVGQEMGDFNSEGIFDVDHATETAEKWLEWNGRTKSNLREEMEECAKITGTGTCMTTYLITKCAMKAGE

>SgreOBP1

WDVNMKLTGRIMDAAKEVDHKCRGSTGVPREMLHRYADGETVDDDDFKCYLKCIMIEFNSLSEDGVFVLEEELENIPPEIKEEGHRVVHSCKHINHDEACQTAYQIHQCYKQSDPELYSLVVRAFDATI

>SgreOBP2

ESPPHMAAFTFTKEMLSTCQEKSEISQEMVDEMQKNKGLLPDESSVAQRCFQECMAKEMGLLKNGGGVAVDNIVTVLKAALQMASEGSEDTYTIDTDAVTRDLEGCQFEGEDDECNNSHDTMKCLRSLGNPENMKRYITKES

>SgreOBP3

MLLAAPAKADEPDLTKAIKDLKDCMASENLDSLDGLKTNKEATTTEEKCFIGCMMKSVHVLNSDGEYDVDFLKEHINHCPELMKDQQKKAAAIEVAESCAAKVTGCSGYCECGVVAGDCMSEGMEAKGYETIYEWLEKVIVKVDA

>SgreOBP4

DPAKLKQAVEKCKASENLDSLDGIKANRQPFTSEEKCFLGCMTLDMKFLSADGQYDAASTKQMINNCEHLKDKPDEKSAALAVADDCGKTVTGCNGYCECGPMTVGCLIKGMMAKGYEESFARIDKVLQKLDG

>SgreOBP5

LSLEQLRQTSKIVRNMCLKKTSVDLALVEGIQEGKFPDDQNLKCYMKCCMGAMQVLRQGRYNVNAAKNQAEKMLPPDLKDRFIAMLDACSDQAVGEDDCEMAYQLTKCSYEADKEIFLFP

>SgreOBP6

QDDDMKEMMEQLHQSCLGESGASDANIDEARKGNFIEDGNLKCYMKCIFVQMTCMSDDGVFDADTAIAMLPDNLKDVASKALTACKDEKGSDACDTAFKINQCLFKQAPKDYILV

>SgreOBP7

RRGPRPFGRCASSVGDIDRDTMMKIVRNFEVPDETSDDQKCMLRCALMTNRLVRDGVLDTRQILMNIRADAHFAGRMASVYGQNITLDMDRLSNDVEACVTSEESPDCDTVYNQFKCVSDLISNGNMASYASFVEVDGSETGDWMRRRQMLASRPHGPPGPMGGHWGPPPPHHRGGPRGGGRPPPPPPESDENDVEELE

>SgreOBP8

MEMTPEFMEIINKCKAEHEPTEDELKGIMMMKVPESEHGKCFMGCVLQEVGVVKDGKFDKEEAKKHAAAKMSDKDELEKHMQLIDKCSQEVDGETDSCGIGPKLMECIKQFAPEFDIALPHAPSE

>SgreOBP9

VLQAPWCPTTASSGVQDDMGLCAEELKDAILREYAKSVAARRTRSAEMSDEDRLLVGCMVSCLFRKGPHGRLQTGSKLALAELGAMRLFSDDAKDARYLNATADAVRRCSAASRSLLPDNGGPRHECELGFFMFECVSDQITEYCQWQKE

>SgreOBP10

EISESMSRAEEAAAKINLPELFEECNETFPIPKVTLNYFFSHGRLQNENDYVAKCFIHCLTDRSGEIDSEGDFDVDLIKVMTRRFPNETNIEGLSEMVDKCVAGRGETDFCERAYGLVSCLVKEKLARLGHSH

>SgreOBP11

DEMLPNTDIPATMAECNATFKLGWRCWDNLLSDGHVIDESKYQQKCWFYCLLDRTGAMHADGAFDKDLLKMVLQGFPNGPSLAHLNETTYTCVAQRSEVDLCERAYAIVKCIMTEELSRMHHSS

>SgreOBP12

LKCHTDEDSQNPDEFQEVAAICMKNTSGSELNRSDRENKRNGNNYHKNNFGNTNDNWSSGGMGQTFPGYNSENEGYGLHGSGRCTANNDGYNNNRNNMNQNNMNGMRQKPRNRNRRSGQQSEAANVDLEDIEPCAVHCIFRQMGMLGDDALPDRSAVAKVMLRGVKDTEVKDFVQEAVEDCFDQVESDRKGSKCDLSKNVALCLRQKGRENCEDWGEQEDDQQSNQNKNGNNNGNNSNNSNNQYGNKKWN

>SgreOBP13

MDKCSFHFCLTNTLIYFSLELSSVLPWITRAEVMKRVNVWTASDELRKKLLDALEECIITENEDLNSSLWSPIKGSPPYGGNTWNIGVATANSNKSINQWRSYNEMMGNRTTSVNRDQKVHIDGVYWKNDSDDQDYKNWKESKCFNRGGNHQMQQRCRRSSELPGGNALSSCVDQCLFVKLQVVDKNGLPVEALFMELLDTSIPEQQMRRKARSELHYCFQKMASVAEEDTCTFGKQFASCLDLNVQDIKKHQSNSSNINKLH

>SgreOBP14

EETFSKNQLKAAVNECNDTYFLSQKSWDSVFTTGSLDDEKDLVAKCFFECVLLQTGAMDDKGTINSDVTKAVFLASHDGTAVDGHGELIDMCVPGRVETDTCEKAYALVKCVTVEELSRRQAR

>ApisOBP1

ESDQVPINSSAAVESCLLETNMTRDEFEDMLTSPNARELTILKSHAHKCMFGCVMRKNHIVNDGVVSKEVLSKYVLNFYGRPDYKRRLIIKDVEHIVDVCAKKVADESETDECELAATLVTCIVLEANKAGLVDDPARQI

>ApisOBP2

SDPCNISTCYKSGTTKPPMAVTPTHLPVQSSSTQTSHPQTTYAKDHVHGSTTTKSGVNATVTTASGASVNGTEPPAVVKSSAGVTGNSTTPKPTMTEGHVALKQKLNTIAVKCKDELHAPQEIMALVSNTVVPQNEQQRCYLECVYKNLNLIKNNKFSVEDGKAMARIRFANQPEEHKKAVTIIETCEKEAVIDPKTTEKCAAGRVIRNCFVKNGEKINFFPKA

>ApisOBP3

RFTTEQIDYYGKACNASEDDLVVVKSYKVPTTETGKCLMKCMITKLGLLNDDGSYNKTGMEAGLKKYWSEWSTEKIESINNKCYEEALLVSKEVVATCNYSYTVMACLNKQLDLDKST

>ApisOBP4

QKQETSGKCRAPDKAPLNLEIIINTCQEEIKSALLQEALDILNDGNVEQNTPNYSSRSKREAEEDLTNEERRVAGCLLQCVYKKVKAVDETGFPVVDGLMKLYNEGVQDRNYYIATLSAVRHCISIAQQLKQQQPSKSFDDGQTCDLAYEMFECVSEKIEENCGVENKSNN

>ApisOBP5

DAGHHRRGKELLDTEDSDFFRCKQASRKSCCGPENAMKRFGDKDKVAADECYAQVAEKFATVTATTPKQDLFSAEAVKITKKKQFCLHECIGKKNNLLTEDGSLNKTFIADYAMKSVFKEQWQKQVGQKALDKCLEETYIPWPAEDKENVCNPVYVQFQHCLWLQYESNCPANKIKITKKCEKTRNRYRMQKSTSN

>ApisOBP6

PNILPNLDSTWEKCFETFKQFKDKPETKEYKEMAHGKEPPCLFQCIFMQSGLTTSDGKLNEDAITKKMSEGINNDEKWKSIWQNSLNKCFDDVKQEDKKQILIMNTPAGRLMKCFLRDMYMSCPKNVWVESSECLNMKDLVQKCPEMPPPVFKSPPKLI

>ApisOBP7

YLSEAAIKKTQQMLKTVCSKKHSVEEDVFTNIKKGIFPEDNNNIKCYFACNFKTMQLINQKGVIDKKMFKDKMSMMAPPNVYKILLPVIEQCTGKDKGEELCQSSYNVIKCAHSVDPKSLEFLPL

>ApisOBP8

ENNQQNGPSDRSATIFQSCIAETKLSGDALKGFRSMSIPKTQAEKCMMGCLMRKVNVINKGKFSVEEATKVAQKYYGTNEAMMKKAKDLIDVCAKKAQSTTEECALAGIVTTCIVEEAQKAGLSGGPGSRSRRTVSPKFRRDAM

>ApisOBP9

DDADAKDKELMSKLFTVVFKCFKDADWGTCGEMITTKYDITQAKYKQCTCHMACAGEELGMINASGQPEPAKFLEYVNKINNPDIKSQLQLIYDKCQNVKGSEKCDLAEQFAICAFKESPALKERVSTLMEMLVKMKPKSK

>ApisOBP10

STRPQPDEMEEIKRTLYNACAGKFPITEEIKNNAKNSIISDDPTFKCFLKCCFDEMSMIDEDGIIDGDSLKAMAPDHIKPILEQVIPSCTKNVKQDGCEASFEFISCGIKLNPLIVALLPL

>ApisOBP11

IFTTEQIDYYGKACNASEDDLIVLKSYKVPSTETGKCLMKCMITKLGLLNDDGSYNKTGMEAGLKKYWSEWATEKIETINEKCYEEGNTATLLYHVAIYFTCVSGDYSDVQLLIHCDGMFEQEVGSRQVNLKLLIMLKIGLSEPKR

>ApisOBP12

DDLVVVKSYKVPTTETGKCLMKCMITKLGLLNDDGSYNKTGMEAGLKKYWSEWSTEKIESINNKCYEEGDTSTLLYHVVIYFTCVKGGSSDVQLLVHCDGMFEQAVGSRQVNL

>ApisOBP13

CTIHCVFNQLEMLNSNSRPDKYSIVNIMTNQIKDVELKEFIQDSIDECFDTLELDSHNNKCEFSKNFAVCMENKAQRNCDDWDENLSANKINSAGLQDGTNQQDKRKGY

>ApisOBP14

TFTTEQIDYYGRACKASEDDLVVVKSYKVPSTETGKCLMKCMITKLGLLNDDGSYNKTGMEAGLKKYWSEWSTEKIEAINNKCYEEALLVPKEIVATCDYSYTVMACLNKQMDLDKLT

>ApisOBP15

MALLIRPKTDGNDAIEEMKNTLYNICSTKYRITEVSKLKNNVKKSIISDDPTFKCFLKCCFDEMSLIDEDGIIDGDSMIQMTSDDDKLIAEQVIPNCIKTVKQDSCEAAFDFVSCVIKLNPSTAALLPL

>ApisOBP16

MENGVTDFTHVSHGNTGSRSKNHRQGSYPSADYDSGQSNSNYNRQSLPTRRYRRDDSNEKSKRQKAAVTGSNNRLLGNNRFRNMTKTGGNQPGKGTYLDKMDACTIHCVFNQLEMLNSNSRPDKYSIVNIMTNQIKDVELKEFIQDSIDECFDTLELDSHNNKCEFSKNFAVCMENKAQRNCDDWDENLSANKINSAGLQDGTNQQDKRKGY

>DmelObp18a

RVNAEGCLKHHNLTSAQVQAVAPSTPVADVPVAVKCYSRCLIQDYFGDDGKIDLQKVGKRGSQEDHVILSQCKQQFDGVTNLDTCDYPYLILQCYFKGKQSGTIAS

>DmelObp19a

GVTEEQMWSAGKLMRDVCLPKYPKVSVEVADNIRNGDIPNSKDTNCYINCILEMMQAIKKGKFQLESTLKQMDIMLPDSYKDEYRKGINLCKDSTVGLKNAPNCDPAHALLSCLKNNIKVFVFP

>DmelObp19b

DEEEGSMTVDEVVELIEPFGDACTPKPSRENIVEMVLNKEDAKHETKCFRHCMLEQFELMPEDQLQYNEDKTVDMINMMFPDREDDGRRIVKTCNEELKAEQDKCEAAHGIAMCMLREMRSSGFKIPEIKE

>DmelObp19c

QTQAFDLAKLLPKTGTEPIWAVIDRNLPQVQELVTAARMECIQKLQLPRDQRPLGKVTNPSEKEKCLVECVLKKIKLMDADNKLNVGQVEKLTSLVTQDNKMAIAVSSSMAQACSRGISSKNPCEVAHLFNQCISRQLERNNVKLVW

>DmelObp19d

KPHEEINRDHAAELANECKAETGATDEDVEQLMSHDLPERHEAKCLRACVMKKLQIMDESGKLNKEHAIELVKVMSKHDAEKEDAPAEVVAKCEAIETPEDHCDAAFAYEECIYEQMKEHGLELEEH

>DmelObp22a

TKEPEEVKIVSECAKENNVHRKKALDLLMSYRLKKKTHNVMCFINCIFERTNILQKVKEKVVKENHNCDSIKDADKCAESFQKFQCLVKIEMKVRGIDRG

>DmelObp28a

FDEKEALAKLMESAESCMPEVGATDADLQEMVKKQPASTYAGKCLRACVMKNIGILDANGKLDTEAGHEKAKQYTGNDPAKLKIALEIGDTCAAITVPDDHCEAAEAYGTCFRGEAKKHGLL

>DmelObp47a

RFAKININLGLTVADESPKTITEEMIRLCGDQTDISLRELNKLQREDFSDPSESVQCFTHCLYEQMGLMHDGVFVERDLFGLLSDVSNTDYWPERQCHAIRGNNKCETAYRIHQCQQQLKQQQQNLLATKEVEVTTTPAGSDETKP

>DmelObp51a

LFESEANECAKKLGITPDYFENFPHSSRVKCFYHCQMEKLEIIANGVVTPFDLKVLNISPESYDKYGVKVKPCLKLSHRDKCELGYLVFQCLKREFNL

>DmelObp56a

SSLNLSDEQKDLAKQHREQCAEEVKLTEEEKAKVNAKDFNNPTENIKCFANCFFEKVGTLKDGELQESVVLEKLGALIGEEKTKAALEKCRTIKGENKCDTASKLYDCFESFKPAPEAKA

>DmelObp56b

QSAAELAAYKQIQQACIKELNIAASDANLLTTDKEVANPSESVKCYHSCVYKKLGLLGDDGKPNTDKIVKLAQIRFSSLPVDKLKSLLTSCGTTKSAATCDFVYNYEKCVVKGISA

>DmelObp56c

KAWVMFFIFYISFTRSLSVSLNMSMTRTLVPDPPNGTENKLSQEMLRACMRRTEISMSQLKLFHMSLMNSDYNNDNDIAPTPVQSIGDVNNLGDLDFNGNSQMPYLDLKHNEPLQCFVSCLYETLDLDRYNVLLEEAFKNQVQTIIQHEKAEIKECSDLQGKTRCEAAYKLHLCYNHLKTLEAEQRIREILERTEAENEGFGPEGSDFIDGIQHSGEAMTTAKSE

>DmelObp56d

ELQLSDEQKAVAHANGALCAQQEGITKDQAIALRNGNFDDSDPKVKCFANCFLEKIGFLINGEVQPDVVLAKLGPLAGEDAVKAVQAKCDATKGADKCDTAYQLFECYYKNRAHI

>DmelObp56e

SAVGLTDSQKAEAKQRAKACVKQEGITKEQAIALRSGNFADSDPKVKCFANCFLEQTGLVANGQIKPDVVLAKLGPIAGEANVKEVQAKCDSTKGADKCDTSYLLYKCYYENHAQF

>DmelObp56f

MKSSEKIKACLKRQLGYTITENTKFDAKEDSLQSKCFYHCLLEVKGVIANDAISSEQPRKVLEKKYGITDTDELEKAEEKCHSIKASGKCELGYEILKCYQSITKH

>DmelObp56g

QQANIDSSVSKELVTDCLKENGVTPQDLADLQSGKVKAEDAKDNVKCSSQCILVKSGFMDSTGKLLTDKIKSYYANSNFKDVIEKDLDRCSAVKGANACDTAFKILSCFQAAN

>DmelObp56h

NPDFRQIMQQCMETNQVTEADLKEFMASGMQSSAKENLKCYTKCLMEKQGHLTNGQFNAQAMLDTLKNVPQIKDKMDEISSGVNACKDIKGTNDCDTAFKVTMCLKEHKAIPGHH

>DmelObp56i

GPIKDQCMAAAGITAQDVANRHETDDPGHSVKCFFRCFLENIGIIADNQIIPGAFDRVLGHIVTAEAVERMEATCNMIKSETSHDESCEFAWQISECYEGVRLSDVKKGQRTRNHRG

>DmelObp57a

KESQPFDFFEGTYDDFIDCLRINNITIEEYEKFDDTDNLDNVLKENVELKHKCNIKCQLEREPTKWLNARGEVDLKSMKATSETAVSISKCMEKAPQETCAYVYKLVICAFKSGHSVIKFDSYEQIQEETAGLIAEQQADLFDYDTIDL

>DmelObp57b

RHPFDIFHWNWQDFQECLQVNNITIGEYEKYARHETLDYLLNEKVDLRYKCNIKCQLERDSTKWLNAQGRMDLDLMNTTDKASKSITKCMEKAPEELCAYSFRLVMCAFKAGHPVIDSE

>DmelObp57c

QSLSLLEETNYVSDCLASNNISQAEFQELIDRNSSEEDDLENTDRRYKCFIHCLAEKGNLLDTNGYLDVDKIDQIEPVSDELREILYDCKKIYDEEEDHCEYAFKMVTCLTESFEQSDEVTEAGKNTNKLNE

>DmelObp57d

MPEKMSLRLVPHLACIIFILEIQFRIADSNDPCPHNQGIDEDIAESILGDWPANVDLTSVKRSHKCYVTCILQYYNIVTASGEIFLDKYYDTGVIDELAVAPKINRCRYEFRMETDYCSRIFAIFNCLRQEILTKS

>DmelObp57e

NPCVSQNELSEYEAHQVMENWPVPPIDRAYKCFLTCVLLDLGLIDERGNVQIDKYMKSGVVDWQWVAIELVTCRIEFSDERDLCELSYGIFNCFKDVKLAAEKYVSISNAK

>DmelObp59a

LKCRSQEGLSEAELKRTVRNCMHRQDEDEDRGRGGQGRQGNGYEYGYGMDHDQEEQDRNPGNRGGYGNRRQRGLRQSDGRNHTSNDGGQCVAQCFFEEMNMVDGNGMPDRRKVSYLLTKDLRDRELRNFFTDTVQQCFRYLESNGRGRHHKCSAARELVKCMSEYAKAQCEDWEEHGNMLFN

>DmelObp69a

VEINPTIIKQVRKLRMRCLNQTGASVDVIDKSVKNRILPTDPEIKCFLYCMFDMFGLIDSQNIMHLEALLEVLPEEIHKTINGLVSSCGTQKGKDGCDTAYETVKCYIAVNGKFIWEEIIVLLG

>DmelObp76a

MTMEQFLTSLDMIRSGCAPKFKLKTEDLDRLRVGDFNFPPSQDLMCYTKCVSLMAGTVNKKGEFNAPKALAQLPHLVPPEMMEMSRKSVEACRDTHKQFKESCERVYQTAKCFSENADGQFMWP

>DmelObp83b

QEPRRDGEWPPPAILKLGKHFHDICAPKTGVTDEAIKEFSDGQIHEDEALKCYMNCLFHEFEVVDDNGDVHMEKVLNAIPGEKLRNIMMEASKGCIHPEGDTLCHKAWWFHQCWKKADPVHYFLV

>DmelObp83g

KFLLKDHADAEKAFEECREDYYVPDDIYEKYLNYEFPAHRRTSCFVKCFLEKLELFSEKKGFDERAMIAQFTSKSSKDLSTVQHGLEKCIDHNEAESDVCTWANRVFSCWLPINRHVVRKVFA

>DmelObp84a

LQDHAKDNGDIFIINYDSFDGDVDDISTTTSAPREADYVDFDEVNRNCNASFITSMTNVLQFNNTGDLPDDKDKVTSMCYFHCFFEKSGLMTDYKLNTDLVRKYVWPATGDSVEACEAEGKDETNACMRGYAIVKCVFTRALTDARNKPTV

>DmelObp99a

ADYVVKNRHDMLAYRDECVKELAVPVDLVEKYQKWEYPNDAKTQCYIKCVFTKWGLFDVQSGFNVENIHQQLVGNHADHNEAFHASLAACVDKNEQGSNACEWAYRGATCLLKENLAQIQKSLAPKA

>DmelObp99b

DHHHHHHDYVVKTHEDLTNYRTQCVEKVHASEELVEKYKKWQYPDDAVTHCYLECIFQKFGFYDTEHGFDVHKIHIQLAGPGVEVHESDEVHQKIAHCAETHSKEGDSCSKAYHAGMCFMNSNLQLVQHSVKV
